# Nitrate availability influences powdery mildew resistance and secondary metabolism in *Gerbera hybrida*

**DOI:** 10.3389/fpls.2026.1879542

**Published:** 2026-08-07

**Authors:** Estuardo J. Hernandez Olesinski, Anna Mascellani Bergo, Tommaso Barbagli, Jaroslav Havlik, Leo F. M. Marcelis, Kirsten A. Leiss

**Affiliations:** 1 Business Unit Greenhouse Horticulture, Wageningen University & Research, Bleiswijk, Netherlands; 2 Department of Food Science, Faculty of Agrobiology, Food and Natural Resources, Czech University of Life Sciences Prague, Prague, Czechia; 3 Horticulture and Product Physiology, Plant Sciences Group, Wageningen University and Research, Wageningen, Netherlands

**Keywords:** ^1^H NMR metabolomics, *Gerbera hybrida*, nitrate fertilization, *Podosphaera xanthii*, secondary metabolism

## Abstract

Powdery mildew (*Podosphaera xanthii*) is a major disease affecting gerbera (*Gerbera hybrida*, Asteraceae), and nitrogen availability is a key factor influencing disease development. Our objective was to determine how nitrate availability affects resistance to *Podosphaera xanthii* through changes in primary and secondary metabolism. Using greenhouse experiments and ^1^H nuclear magnetic resonance-based metabolomics, we found that lower nitrate levels reduced infection, supporting the concept of nitrogen-induced susceptibility. Reduced nitrate availability was associated with decreased amino acid levels and increased accumulation of defense-associated metabolites such as polyketide and phenylpropanoid metabolites, including gerberin, gerberinside, parasorboside, and chlorogenic acid derivatives. Notably, moderate nitrate reduction enhanced resistance without compromising flower biomass, provided that nitrate supply remained above 3 mM. Our results highlight nitrate management as a potential strategy for sustainable disease control.

## Introduction

Nitrogen fertilization plays a pivotal role in plant growth and development, and shapes complex interactions between plants and pathogens. Nitrogen impacts both host defense mechanisms and pathogen virulence (Sun et al., 2020). Furthermore, the specific form of nitrogen supplied plays a decisive role in pathogen development (Snoeijers and Perez-Garcia, 2000). Higher nitrate (NO_3_^−^) supply has been linked to greater incidence of biotrophic pathogens, including those responsible for powdery mildew, *Colletotrichum*, and rust diseases. In contrast, higher ammonium (NH_4_^+^) supply tends to decrease their incidence (Dordas, 2008; Fagard et al., 2014; Hofer et al., 2016). Conversely, an increase in ammonium and a decrease in nitrate enhances the incidence of necrotrophic and hemibiotrophic pathogens such as *Phytophthora* and *Fusarium* (Solomon et al., 2003; McGovern, 2015).

The influence of nitrogen form on plant-pathogen interactions is mediated through complex metabolic and signaling processes. Variations in nitrogen source and availability can lead to shifts in primary metabolism, amino acid and organic acid profiles, and key defense pathways involving salicylic acid, nitric oxide, and other signaling compounds (Mur et al., 2016; Thalineau et al., 2016; Ding et al., 2021). A recent study in rice further demonstrated that nitrogen-induced susceptibility has a genetic component and may be associated with differences in nitrogen assimilation and the ability to rapidly activate defense responses, including peroxidase activity and lignin biosynthesis (Liu et al., 2026). Nitrogen nutrition can also modify carbohydrate metabolism, including the balance among sucrose, glucose, and fructose. These changes can modulate the synthesis of antimicrobial compounds and ultimately alter plant resistance to pathogens (Lecompte et al., 2013; Yin et al., 2020; Lacrampe et al., 2021).

Gerbera (*Gerbera hybrida*, Asteraceae) is a globally important ornamental crop, ranking among the top five cut flowers in terms of economic value (Teeri et al., 2006). In the Netherlands, greenhouse production of gerbera covers an area of 108 hectares (CBS StatLine, 2026). Beyond its commercial importance, gerbera is also a useful model for studying flower development and secondary metabolism. In particular, it has been used in studies of anthocyanin and polyketide biosynthesis (Teeri et al., 2006; Ruokolainen et al., 2010; Bashandy et al., 2015). However, gerbera production faces major challenges from powdery mildew, primarily caused by *Podosphaera xanthii* (Kloos et al., 2005). This obligate biotrophic fungus forms characteristic white mycelial patches on leaves and stems, severely impairing photosynthesis, plant vigor, and aesthetic quality (Song and Deng, 2013; Zhang et al., 2021). The resulting crop losses and increased management costs impose a substantial economic burden on growers. Although certain gerbera cultivars exhibit partial resistance, current control strategies rely heavily on chemical fungicides, raising concerns about sustainability and resistance development (Tanaka et al., 2017; Ghani and Sharma, 2019; Mascellani et al., 2022).

In our previous study, we identified resistance-associated metabolites in several *Gerbera hybrida* cultivars, including gerberin, parasorboside, gerberinside, and 5-hydroxyhexanoic acid 3-O-β-D-glucoside (Mascellani et al., 2022). Given that nitrogen availability strongly influences secondary metabolism (Mur et al., 2016), the present study examines whether nitrate availability is associated with changes in these metabolites and with powdery mildew susceptibility.

The aim of this study was to determine how nitrate availability influences gerbera resistance to powdery mildew by affecting primary and secondary metabolism. We hypothesized that lower nitrate supply enhances resistance by promoting the accumulation of secondary metabolites associated with pathogen defense. To address this hypothesis, we formulated the following research questions: (1) Does a lower nitrate supply reduce the severity of powdery mildew infection in gerbera? (2) Is a reduction in disease severity associated with changes in secondary defense metabolites derived from the polyketide pathway? (3) If lower nitrate enhances resistance, does it also compromise plant growth, yield, or flower quality?

## Materials and methods

### Experimental site and cultivation

Two trials were performed in a Venlo-type glasshouse compartment (12 × 10 m; 120 m²) in 2021 (23 March–1 July, addressed as Trial 1 throughout the study) and 2022 (7 April–1 July, addressed as Trial 2 throughout the study) using *Gerbera hybrida* ‘Kimsey’ plants. One month old gerbera seedlings, previously grown in stone wool plugs and irrigated with a standard nutrient solution for seedlings, were transplanted into 7 L plastic pots filled with granulated stone wool. The crop was managed following standard greenhouse protocols, with flowers harvested approximately twice per week.

Each compartment was equipped with 35 PVC boxes (270 L; 1.02 × 0.64 × 0.55 m) containing the nutrient solution. Six pots were placed on the lid of each box. The path between the boxes was 0.9 m wide. The plant density was 3.3 plants m^-2^. Each box included a water pump (Eden 130G), distributing nutrient solution through six capillaries (one dripper per plant). Drainage from the pots returned to the box through holes in the lid and was recirculated. Irrigation was scheduled via climate computers with time and radiation-dependent triggers. Climate conditions were maintained at 22 °C (day) and 19 °C (night), with CO_2_ concentrations of 620 ppm (day) and 567 ppm (night), and relative humidity of 60% (day) and 75% (night).

### Nutrient treatments

In the first trial, five NO_3_^−^ levels were tested (**Table 1**). In the second trial, four intermediate NO_3_^−^ concentrations were evaluated (**Table 1**). The highest NO_3_^−^ concentration (19 mM NO_3_^−^) was the concentration that is typically used in commercial greenhouse production of gerbera. In all treatments, the electrical conductivity (EC) was kept constant at 2.3 dS m^-1^ and pH at 5.7. Decreasing NO_3_^−^ was compensated by proportionally increasing SO_4–_^2^ and Cl^-^ (**Table 1**). For the standard nutrient solution, the concentrations of the other ions were NH4^+^, 0.5; K^+^, 5.9; Ca^2+,^ 5.9; Mg^2+^, 2.3; and P (as phosphate), 1.2 mM; Fe, 25; Mn^2+^, 15; Zn^2+^, 5; B (as boric acid/borate), 25; Cu^2+^, 1; and Mo (as molybdate), 0.5 μM. Treatments were applied from transplanting onward.

**Table 1:** Average achieved concentrations of the major anions in the nutrient solution of trial 1 and 2.

Trial	Treatment label	NO_3_^-^(mM)	SO_4_^-2^ (mM)	Cl^-^ (mM)	H_2_PO_4_ (mM)
**1**	2 mM NO_3_^−^	1.8	6.6	6.6	1.2
	3 mM NO_3_^−^	3.2	6.2	6.1	1.2
	5 mM NO_3_^−^	4.7	5.6	5.6	1.2
	8 mM NO_3_^−^	7.9	4.6	4.6	1.2
	19 mM NO_3_^−^	19.0	1.3	0.0	1.2
**2**	3 mM NO_3_^−^	3.0	6.2	6.2	1.2
	5 mM NO_3_^−^	5.0	5.5	5.5	1.2
	7 mM NO_3_^−^	7.0	4.9	4.9	1.2
	10 mM NO_3_^−^	10.0	3.9	3.9	1.2
For both trials, EC was 2.3 dS m^-1^ and pH 5.7					

### Nutrient solution measurements and sampling

Fresh weight of flowers was recorded at each harvest. Mean flower biomass was calculated per nutrient box (experimental unit). At the end of the trial (day 92), one representative plant per box was harvested. Approximately one quarter of the aboveground biomass from each replicate was dried at 80 °C for one week and ground. Total nitrogen analysis was performed by Groen Agro Control. In Trial 1, samples from individual replicate boxes were analyzed separately (n = 3 per treatment), whereas in Trial 2, leaf material from the six replicate boxes was pooled prior to analysis, resulting in one composite sample per treatment.

Nutrient solutions from the boxes were collected biweekly and pooled per treatment. One sample per treatment was sent biweekly to laboratory Groen Agro Control for nutrient analysis. EC, pH, and NO_3_^−^ were also measured weekly manually (Hanna HI9813-51; Horiba LaquaTwin NO_3_) (**Supplementary Figures 1**, **3**). All nutrients (including micronutrients) were adjusted individually when needed. pH was maintained between 4.5 and 6.3 using KOH or H_2_SO_4_. Supply and drain water volumes were recorded daily in at least one box (**Supplementary Figures 2**, **4**). From this observation, the drain percentage was calculated as the ratio between the drain and supply volume.

### Powdery mildew bioassays

Powdery mildew bioassays were conducted at the end of the vegetative growth phase using leaf discs. The youngest fully expanded mature leaf of each plant was sampled, and 5 cm diameter discs were excised using a cork borer. Each disc was placed abaxial side up on 1% (w/v) water agar in a Petri dish. *Podosphaera xanthii* inoculum was obtained from an in-house greenhouse compartment dedicated to the continuous maintenance of the isolate. The isolate was originally collected from naturally infected *Gerbera hybrida* plants in a commercial greenhouse in South Holland, the Netherlands. To preserve infectivity, the isolate was continuously propagated on susceptible gerbera plants in a 25 m² air-conditioned greenhouse compartment maintained at approximately 22 °C and 80% relative humidity. Fresh conidia from actively sporulating colonies were used for inoculations. Discs were inoculated with *Podosphaera xanthii* at a concentration of 1 × 10^5^ conidia mL^−^¹ using a fine atomizer for even spore distribution. The inoculated Petri dishes were incubated in a climate chamber at 20 °C, 60% relative humidity, and a 16 h light/8 h dark photoperiod. Ten days post inoculation, the number of visible powdery mildew colonies per leaf disc was counted to assess infection severity.

### Metabolomics analysis: sample preparation, data preprocessing and annotation

All chemicals and reagents used were of analytical grade. Potassium dihydrogen phosphate (99%, KH_2_PO_4_), deuterium oxide (99.9%, D_2_O), and methanol-*d_4_* (>99.8%, MeOD) were purchased from VWR (Radnor, PA, United States). Sodium deuteroxide 40% w/v solution in D_2_O (99.5%, NaOD) was obtained from Alfa Aesar (Kandel, Germany). 3-(Trimethylsilyl) propionic-2,2,3,3-*d_4_* acid sodium salt (99%, TSP) was obtained from Sigma-Aldrich (St. Louis, MO, United States).

For metabolomic analyses, each biological sample consisted of a fully expanded mature leaf adjacent to the leaf used for the powdery mildew bioassay, collected from the same plant. Trial 1 comprised 18 biological samples per treatment. Trial 2 initially comprised 18 biological samples per treatment; however, three samples could not be processed, resulting in 15 biological samples for data analysis. Each 40 (± 1) mg of the finely ground leaves were extracted in MeOD-D_2_O (1:1, v/v) as previously described (Kim et al., 2010; Mascellani et al., 2021). All spectra were recorded on a Bruker Avance III spectrometer equipped with a broad band fluorine observation (BBFO) SmartProbe™ with z-axis gradients (Bruker, Billerica, Massachusetts, United States), operating at the ^1^H NMR frequency of 500.18 MHz. The ^1^H NMR parameters for spectral acquisition were employed as outlined by Mascellani et al. (2021). Samples were prepared and measured in a random order. Spectra quality was assessed based on symmetry of TSP signal and peak width lower than 1 Hz. The spectra were pre-processed and reduced with an in-house script under MATLAB^®^ R2022a (MathWorks, Natick, MA, USA) consisting of multipoint baseline correction in user-defined segments, ensuring the same pre-processing for all the spectra (Jenickova et al., 2023). Spectra between δ_H_ 0.5 and 9.0 ppm were reduced into defined buckets, with each bin representing a spin system or a part of a spin system that was ideally pure, distinct, and quantitative—one bin for each metabolite. The regions corresponding to water (δ_H_ 4.70–5.00 ppm) and methanol (δ_H_ 3.28–3.40 ppm) were excluded from the analysis. The bins’ ranges were chosen after annotating superimposed spectra (**Supplementary Table 1**) according to 2D NMR spectra and (Leiss et al., 2009; Mascellani et al., 2022). For statistical processing, buckets were normalised to the area of TSP and adjusted to the amount of sample extracted.

### Experimental design and statistical analysis

Two independent greenhouse trials were conducted in 2021 and 2022. Each trial followed a completely randomized design with three (2021) and six (2022) replicate nutrient boxes per nitrate treatment. As the nutrient solution composition was shared within each box, the experimental unit was defined as the box. Boxes were randomly assigned to positions within the compartment, and treatments were applied from the time of transplanting onward.

Data on total biomass and nitrogen content were analyzed using one-way ANOVA followed by Tukey’s *post hoc* test. Powdery mildew bioassay data were analyzed using a generalized linear model (GLM) with nitrate level included as a fixed factor. Estimated marginal means (EMMs) were calculated, and pairwise comparisons among nitrate levels were performed to assess treatment differences. These analyses were performed in SPSS Statistics version 30.0.0 (IBM Corp., 2024). Data from NMR metabolomics were subjected to principal component analysis (PCA) and one-way ANOVA followed by Tukey’s *post hoc* test, with p-values adjusted using the Benjamini–Hochberg procedure. Analyses were conducted in R version 4.4.2 (R Core Team, 2024). PCA was performed using the factoextra package (Kassambara and Mundt, 2020), and average metabolite abundances were visualized in a heatmap with Euclidean distance clustering using pheatmap v1.2.12 (Kolde, 2019).

## Results

### Effect of nitrate concentration on total fresh flower biomass

**Figure 1:**
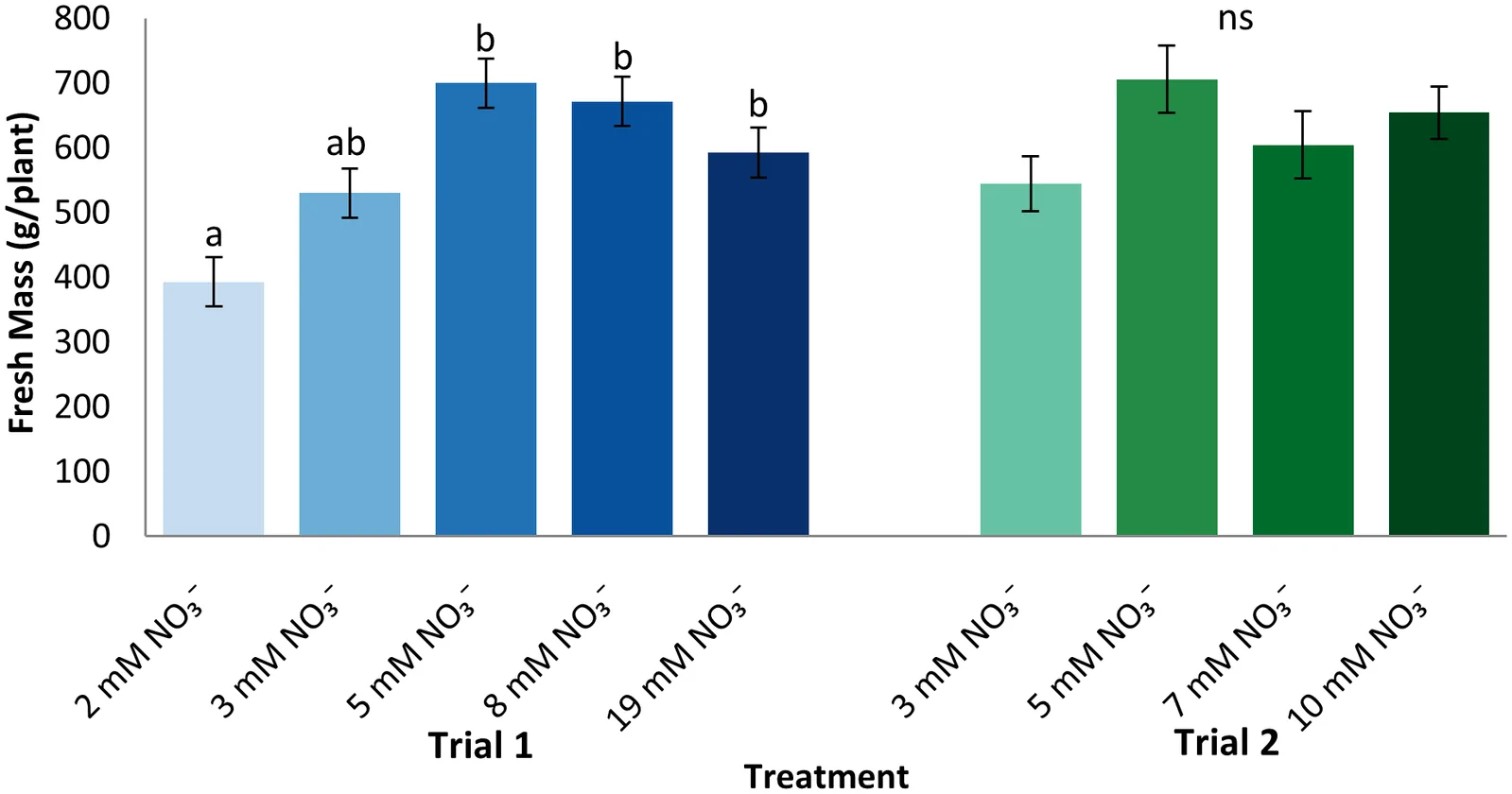
Effect of nitrate concentration on total fresh flower biomass. Different letters indicate significant differences, p = 0.05. Mean values with standard error are shown. Trial 1, n=3; Trial 2, n=6. ns, not significant.

In the first trial employing nitrate concentrations from 2 to 19 mM NO_3_^−^, the total flower biomass was reduced at the lowest nitrate level (2 mM NO_3_^−^), while there were no significant differences in flower biomass among the four higher concentrations (3, 5, 8, and 19 mM NO_3_^−^) (**Figure 1**). In the second trial, employing nitrate concentrations of 3 to 10 mM NO_3_^−^, no significant differences were observed (**Figure 1**).

### Effect of nitrate supply on total nitrogen content

**Figure 2:**
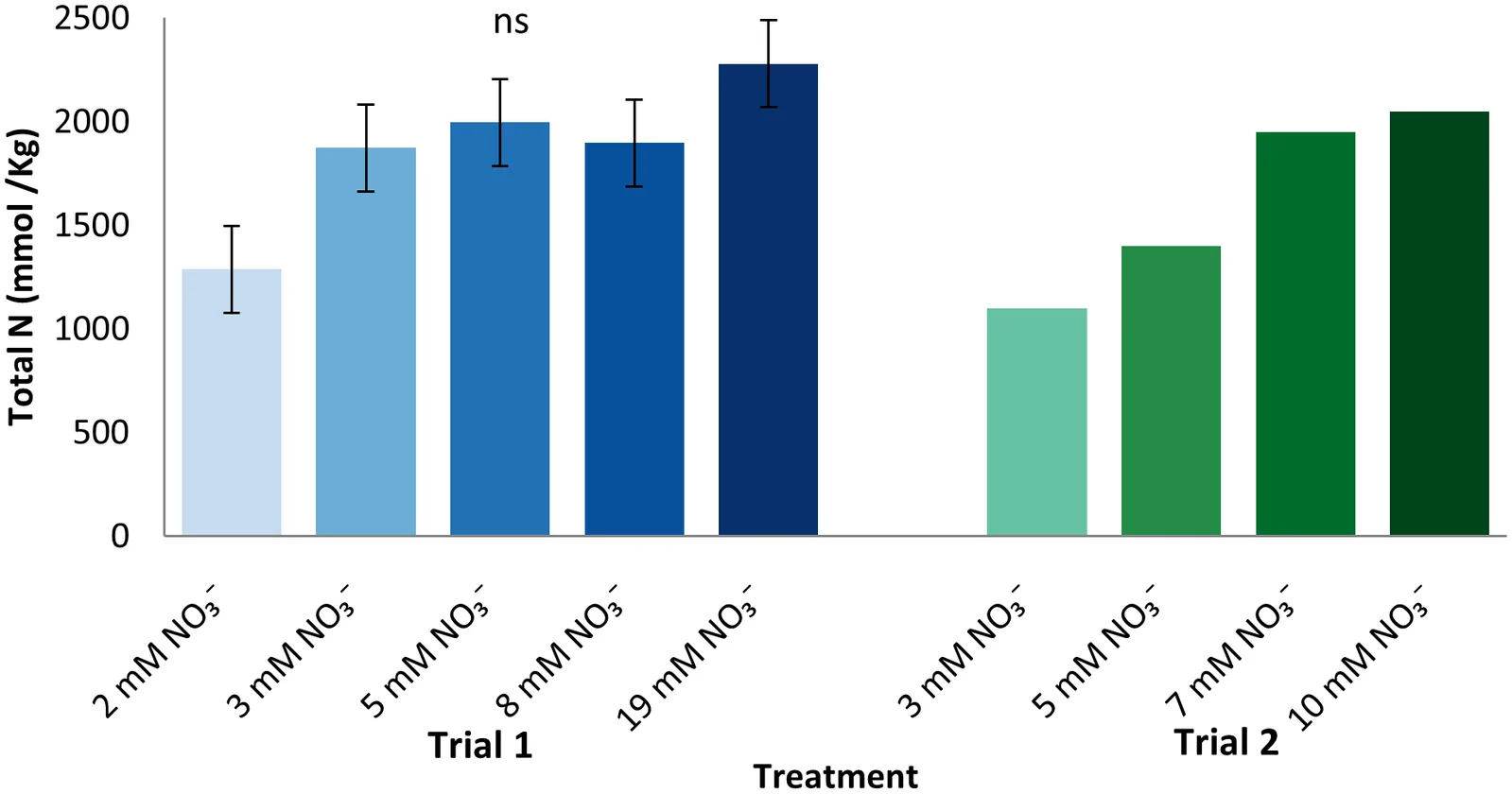
Effect of nitrate concentration in the fertigation water on total nitrogen (sum of organic and inorganic nitrogen) content of the leaves. Values are means ± SE. Trial 1: n = 3. Trial 2: n = 1. ns, not significant.

In the first trial, total nitrogen content in plant tissue did not differ significantly among treatments. However, descriptive comparison of average values in the first trial revealed a difference of 992 mmol/kg between the highest (19 mM NO_3_^−^) and lowest (2 mM NO_3_^−^) nitrogen treatments (**Figure 2**).

### Effect of nitrate concentration on powdery mildew bioassays

**Figure 3:**
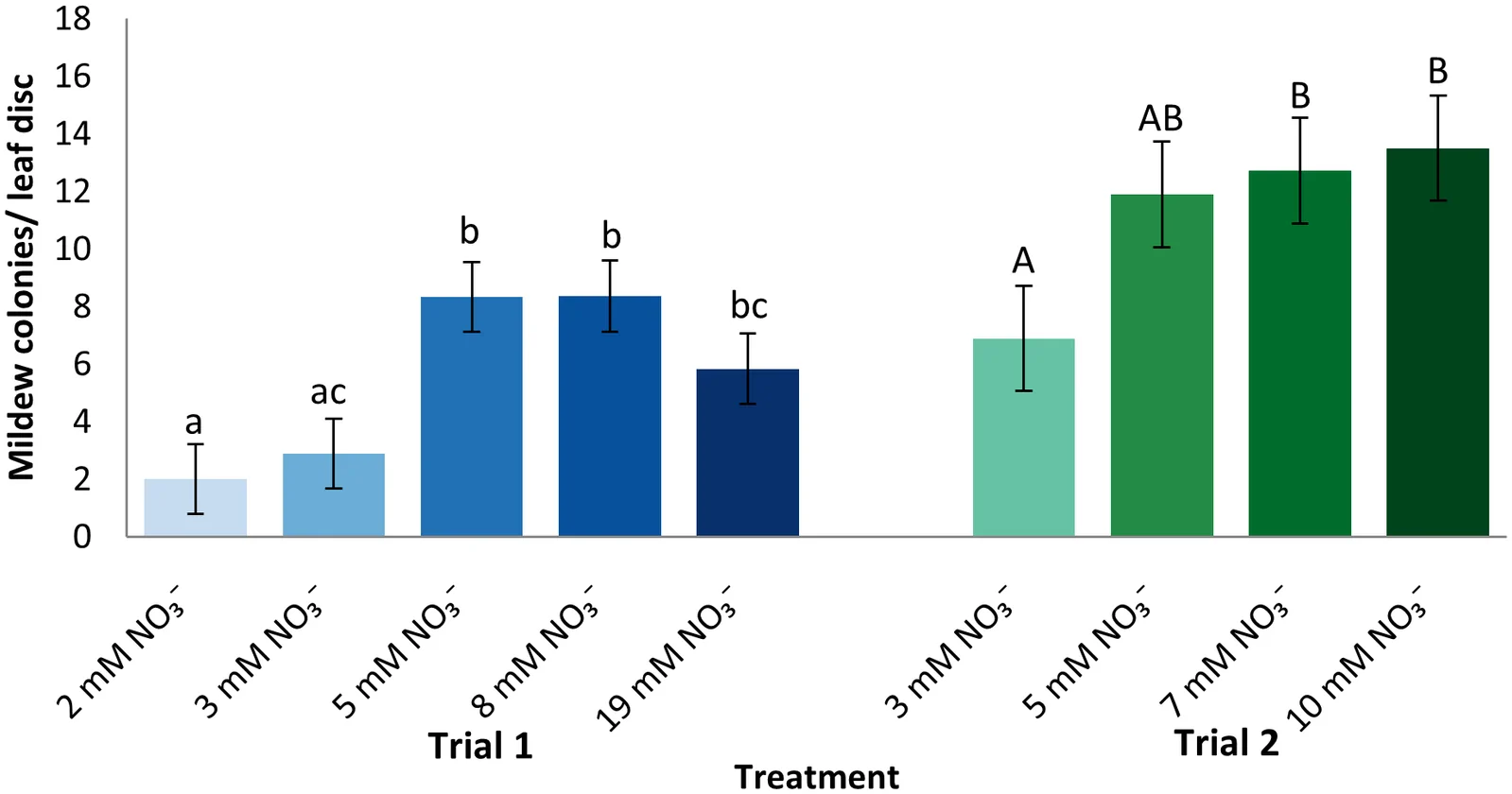
Effect of nitrate concentration on powdery mildew. Different letters indicate significant differences, p = 0.05. Mean values with standard error are shown. Trial 1, n=18; Trial 2, n=18.

A clear trend of reduced powdery mildew infection was observed with decreasing nitrate levels during the generative phase. In the first trial, a significant reduction in the number of powdery mildew spots was detected between higher nitrogen treatments (5 and 8 mM NO_3_^−^) and lower levels (2 and 3 mM NO_3_^−^) (**Figure 3**). Higher nitrogen availability was associated with greater susceptibility to powdery mildew. In the second trial, a similar pattern emerged, with a significant difference between higher nitrogen levels (7 and 10 mM NO_3_^−^) and 3 mM NO_3_^−^.

### Metabolic changes in relation to different levels of nitrate

Sixteen metabolites were identified using a semi-targeted approach covering primarily molecules of polyketides, cinnamic acids, amino acids, and carbohydrates. Additionally, few more signals were included in the analysis as unknown compounds (**Supplementary Table 1**).

**Figure 4:**
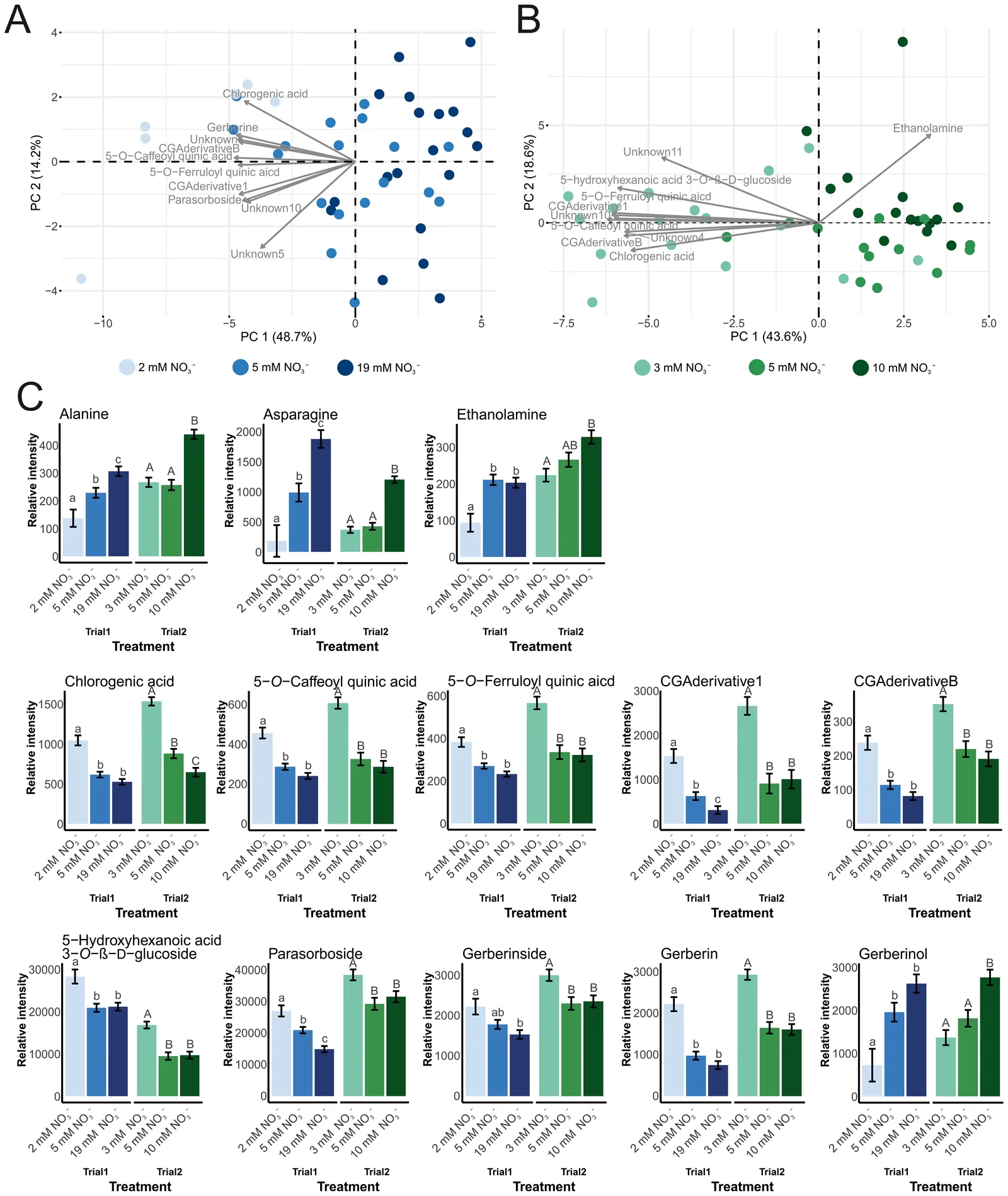
Metabolic changes in relation to different levels of nitrate. **(A)** PCA biplot of metabolites in *Gerbera hybrida* leaves extracted in MeOD-D_2_O (1:1, v/v) from samples collected during Trial 1. The 10 most influential loadings are shown. **(B)** PCA biplot of metabolites in *Gerbera hybrida* leaves extracted in MeOD-D_2_O (1:1, v/v) from samples collected during Trial 2. The 10 most influential loadings are shown. **(C)** Bar plots of selected metabolites. Different letters indicate significant differences (*p* < 0.05) after Benjamini–Hochberg correction. Values are presented as means ± SE. Trial 1, n=18. Trial 2, n=15. CGA, chlorogenic acid.

Metabolic changes were recorded at the generative stage under the different nutritional levels in both trials. The PCA biplot illustrates the relationships between the three nitrate treatments in the first trial (2 mM NO_3_^−^, 5 mM NO_3_^−^, 19 mM NO_3_^−^) and various metabolites along the first and second principal components, which explain 48.7% and 14.2% of the variance, respectively (**Figure 4A**). Comparable clustering was presented using clustering methods (**Supplementary Figure 5**). The plants grown in 2 mM NO_3_^−^ occupied the left-hand side of the score plot, whereas those grown in 19 mM NO_3_^−^ are located predominantly on the right-hand side, indicating a clear separation along PC1. Plants grown in 5 mM NO_3_^−^ showed an intermediate distribution between the two treatments. The same associations were observed in Trial 2, where the PCA biplot illustrates the relationships between the groups (3 mM NO_3_^−^, 5 mM NO_3_^−^, 10 mM NO_3_^−^) along the first two principal components, which explain 43.6% and 18.6% of the variance, respectively (**Figure 4B**). While leaves from plants grown in 10 mM NO_3_^−^ and 5 mM NO_3_^−^ have overlapping profiles, growth in 3 mM NO_3_^−^ was more distinct. Metabolites such as alanine, asparagine, and ethanolamine decreased with decreasing nitrate concentration in the nutrient solution, also confirmed by univariate analysis (**Figure 4C**). On the other hand, gerberin, gerberinside, parasorboside, 5-hydroxyhexanoic acid 3-O-*β*-D-glucoside, and chlorogenic acid derivatives were higher in leaves of plants grown under low nitrate levels (**Figure 4C**). Notably, gerberinol showed an opposite trend, decreasing with lower nitrate levels (**Figure 4C**). Furthermore, other unidentified compounds increased at lower nitrate levels (**Supplementary Figure 6**).

## Discussion

### Reduced powdery mildew susceptibility under lower nitrate supply

Lower nitrate availability markedly decreased the susceptibility of *Gerbera hybrida* to powdery mildew caused by *Podosphaera xanthii*. Plants grown under low nitrate conditions consistently developed fewer colonies, indicating enhanced resistance compared to plants supplied with higher nitrate levels. This observation is consistent with previous reports of reduced powdery mildew severity under low nitrogen availability in wheat and strawberry (Xu et al., 2013; Luo et al., 2021). However, these earlier studies primarily described phenotypic outcomes without investigating the underlying metabolic changes.

The present results support the concept of nitrogen-induced susceptibility (NIS), in which higher nitrogen availability enhances plant vulnerability to disease, whereas lower nitrogen availability reduces disease severity (Huang et al., 2017). NIS has been documented for multiple obligate biotrophic pathogens, including those causing downy mildew, powdery mildew, gray mold, leaf rust, and stem rot (Bueschbell and Hoffmann, 1992; Tompkins et al., 1992; Howard et al., 1994; Jensen and Munk, 1997; Huang et al., 2017). Our findings indicate that this relationship also applies to *Gerbera hybrida* and suggest that nitrate availability is associated with metabolic changes relevant to plant defense. Powdery mildew resistance was evaluated using a detached leaf-disc assay. This approach has been developed as a standardized and reproducible system for experiments at greenhouse scale to quantify disease development, although it may not fully capture whole-plant resistance.

### Nitrate limitation is associated with enhanced secondary defense metabolism

Reduced powdery mildew severity under lower nitrate supply was accompanied by shifts in primary and secondary metabolism. Nitrate availability was a major factor shaping the leaf metabolome of *Gerbera hybrida*. Across both trials, the lowest nitrate treatments consistently displayed the most distinct metabolic profiles. Although absolute nitrate concentrations varied across experiments, the similar clustering patterns indicate a robust, reproducible metabolic response to nitrate limitation. In particular, alanine, asparagine, and ethanolamine decreased with decreasing nitrate concentration, consistent with reduced nitrogen assimilation and a lower abundance of nitrogen-containing primary metabolites. Asparagine is especially relevant in this context because it represents a major transport and storage form of nitrogen in plants, and its reduction likely reflects constrained nitrogen availability within leaf tissue (Postles et al., 2016; Iqbal et al., 2022; Sakuraba, 2022).

Although total leaf nitrogen did not differ significantly among treatments, this parameter represents the overall nitrogen pool and may not fully capture changes in nitrogen metabolism. Plants can maintain relatively stable tissue nitrogen concentrations through the tight regulation of nitrogen uptake, transport, and remobilization. Nevertheless, nitrate availability continues to influence nitrogen assimilation and metabolic partitioning (Nacry et al., 2013; Feng et al., 2020). Nitrogen-containing primary metabolites, including alanine and asparagine, decreased under reduced nitrate supply despite relatively constant total leaf nitrogen. These observations indicate that metabolic reprogramming in response to nitrate availability can occur before measurable changes in bulk tissue nitrogen become apparent. The total nitrogen measurements obtained in Trial 2 were consistent with the trend observed in Trial 1. Trial 2 was largely a repetition of Trial 1 to verify if observed effects could be repeated. Therefore, in Trial 2, leaf material from the six replicate boxes was pooled prior to total nitrogen analysis, resulting in one composite sample per treatment (n = 1).

In parallel, metabolomic profiling revealed increased accumulation of gerberin, gerberinside, parasorboside, and chlorogenic acid derivatives. These metabolic signatures closely resemble those reported by Mascellani et al. (2022), who found that *Gerbera hybrida* cultivars with constitutively higher levels of polyketide-derived metabolites exhibited lower susceptibility to powdery mildew. Similarly, chlorogenic acid has been shown to directly contribute to resistance to powdery mildew in miniature roses (Shetty et al., 2011). At the same time, gerberinol decreased under low nitrate supply, in contrast to gerberinside, gerberin, parasorboside, and chlorogenic acid derivatives. This finding suggests that nitrate limitation did not uniformly stimulate secondary metabolism. Instead, it altered metabolic partitioning within acetate–malonate-derived specialized metabolism (Jangpangi et al., 2025).

In *Gerbera hybrida*, gerberinside belongs to the 4-hydroxy-5-methylcoumarin branch, whose biosynthesis requires two polyketide synthases, G2PS1 and G2PS2; 4-hydroxy-5-methylcoumarin is subsequently stored as its glucoside, gerberinside (Nagumo et al., 1989; Pietiäinen et al., 2016). By contrast, gerberin and parasorboside are defense-related polyketides whose synthesis is initiated by G2PS1 and further shaped by polyketide reductases such as GRED1 and GRED2 (Zhu et al., 2022). Gerberinol, in turn, has been described as a dimethyl dicoumarol from *Gerbera* spp (Brahmachari et al., 2017). Its decrease under low nitrate may therefore indicate that nitrate limitation shifted metabolic partitioning away from one coumarin-derived metabolite pool and toward glycosylated coumarins and defense-related polyketides. Although gerberinol could hypothetically derive from a phenylpropanoid-related coumarin pathway, there is currently no evidence for this in gerbera, and such an interpretation remains speculative.

The accumulation of these compounds under nitrate limitation may reflect a reallocation of carbon skeletons from nitrogen-rich primary metabolism toward carbon-based secondary metabolism. Under reduced nitrogen availability, carbon skeletons that would otherwise support amino acid and protein synthesis may instead be redirected toward the biosynthesis of nitrogen-free defense metabolites. Comparable increases in chlorogenic acid under low nitrogen supply have been reported in tomato and *Chrysanthemum morifolium*, where the response was attributed to diversion of carbon from primary metabolism to secondary metabolite production (Liu et al., 2010; Bénard et al., 2011).

Consistent with this interpretation, there is currently no evidence that specific stress conditions directly activate the branch of the polyketide pathway responsible for parasorboside or gerberin-related compounds. Most studies linking stress responses to polyketide metabolism instead focus on flavonoid biosynthesis. Although flavonoid production requires enzymes of the type III polyketide synthase (PKS) superfamily, it involves a different metabolic branch than the one examined in the present study (Zhu et al., 2022). Chalcone synthase (CHS), the best-characterized type III PKS, is induced by UV radiation, wounding, and bacterial or fungal infection. This induction promotes the accumulation of defense-related flavonoids and phytoalexins (Austin and Noel, 2003; Dao et al., 2011). In *Marchantia polymorpha*, one member of the CHS gene family is significantly upregulated under UV-B stress (Bowman et al., 2017; Clayton et al., 2018). In addition, knockout mutants of the transcription factor *MpMYB14* show impaired CHS induction under nitrogen deficiency (Kubo et al., 2018). These findings demonstrate stress-responsive regulation of the flavonoid branch of type III PKS metabolism but provide no direct evidence for activation of the distinct polyketide branch associated with parasorboside or gerberin biosynthesis.

Beyond *Gerbera hybrida*, metabolomic studies in other crop species provide further evidence linking metabolic reprogramming to disease resistance. Enhanced resistance has frequently been associated with the accumulation of specialized defense metabolites and the activation of defense-related pathways (Mhlongo et al., 2018). Resistance to Magnaporthe oryzae in rice was associated with increased accumulation of phytoalexins and metabolites from the flavonoid, phenylpropanoid, and polyketide pathways (Iqbal et al., 2025). In onion, induced resistance to Alternaria porri involved distinct antioxidant enzyme responses and was enhanced by salicylic acid treatment (Asghar et al., 2025). Likewise, reduced nitrogen availability increased chlorogenic acid, flavonoids, and other defense-associated metabolites in tomato and Chrysanthemum morifolium (Bénard et al., 2011; Liu et al., 2010), whereas lower nitrogen supply was associated with reduced powdery mildew severity in wheat (Luo et al., 2021).

Reductions in nitrate in the nutrient solution were compensated by proportional increases in SO_4_²^−^ and Cl^−^ as it is not possible to change the concentration of only one ion in a nutrient solution. This raises the question of whether these ions could also have contributed to the observed increase in resistance. Both sulfate and chloride have been reported to reduce powdery mildew development (Kettlewell et al., 2000; Don et al., 2010; Jia et al., 2024). However, chloride-mediated suppression has only been demonstrated through osmotic inhibition of spore germination when applied as foliar sprays at concentrations far exceeding physiological tissue levels (Kettlewell et al., 2000; Don et al., 2010). Sulfur nutrition has been associated with enhanced disease resistance through its role in sulfur metabolism and redox regulation, particularly when elemental sulfur is applied to the soil rather than supplied through fertigation (Jia et al., 2024). The individual contributions of nitrate, sulfate, and chloride could not be distinguished in the present study. Because of the requirement to maintain electrical neutrality, disentangling their respective effects would require a substantially more complex experimental approach beyond the scope of this study.

The increased accumulation of polyketide-derived and phenylpropanoid metabolites under nitrate limitation likely represents a major factor underlying the reduced powdery mildew susceptibility observed in this study. However, nitrogen availability may also influence disease development through additional physiological mechanisms that reinforce this response. Lower leaf nitrogen concentrations have been associated with reduced powdery mildew severity in wheat (Luo et al., 2021), consistent with our observation that plants receiving lower nitrate supply exhibited reduced powdery mildew incidence. Lower nitrogen availability has been associated with reduced pathogen infection through decreased apoplastic amino acid availability, reduced tissue succulence, and canopy structures that are less favorable for pathogen development (Sun et al., 2020; Jeridi et al., 2025). Likewise, the reduced amino acid levels observed under low nitrate supply may indicate decreased nutrient availability for the pathogen. Reduced nitrogen supply has also been associated with increased accumulation of multiple defense-related secondary metabolites (Sun et al., 2020), consistent with the broader metabolic shifts observed here.

Although these metabolic changes coincided with reduced powdery mildew severity, the present study does not establish a direct causal relationship. Several nitrate-responsive processes could independently influence both metabolite accumulation and disease susceptibility. Altered redox status or shifts in reactive oxygen species signaling under nitrate limitation could drive both the observed metabolic changes and the reduction in disease susceptibility (Mur et al., 2016). Changes in phytohormone signaling may also contribute to these responses, particularly through jasmonic acid and salicylic acid. Both pathways are influenced by nitrogen status and play central roles in defense against biotrophic pathogens (Mur et al., 2016; Ding et al., 2021). In addition, reduced nitrate reductase activity may alter nitric oxide homeostasis, thereby influencing both defense gene expression and secondary metabolite biosynthesis (Mur et al., 2016; Thalineau et al., 2016). Alterations in the carbon-to-nitrogen ratio may also contribute to these responses (Liu et al., 2010; Bénard et al., 2011). Furthermore, nitrate limitation may influence disease susceptibility through changes in tissue succulence and host nutritional status that affect pathogen development independently of soluble defense metabolites (Sun et al., 2020; Jeridi et al., 2025). Future studies manipulating individual metabolites or their biosynthetic pathways will be required to determine their direct contribution to powdery mildew resistance in *Gerbera hybrida*.

### Resistance under low nitrate occurs with limited trade-offs for fresh flower biomass

A key question is whether enhanced resistance under nutrient limitation imposes a measurable penalty on plant growth and flower yield. In the present study, nitrate supply affected plant performance in a concentration-dependent manner: flower biomass reduction was statistically significant only at the most restrictive nitrate level (2 mM NO_3_^−^), whereas moderate nitrate reduction maintained flower biomass. This response is consistent with the growth-defense trade-off framework described by Huot et al. (2014) in the *Arabidopsis*–*Pseudomonas syringae* pathosystem, where increased defensive investment under nutrient limitation results in growth penalties only under severe restriction.

Total leaf nitrogen remained relatively stable across treatments, exhibiting only a slight and non-significant decline with decreasing nitrate supply in the first trial. These subtle changes may nevertheless have been sufficient to activate secondary metabolism without markedly affecting biomass accumulation, except under the lowest nitrate condition. This response likely reflects the tightly regulated nature of nitrogen uptake. Root nitrogen acquisition is an energy-dependent process governed by plant demand and systemic signaling. Multiple transport systems allow plants to maintain nitrogen uptake across a broad range of external concentrations (Nacry et al., 2013; Feng et al., 2020).

### Implications for ornamental crop production and disease management

From an applied perspective, these results indicate that nitrate management can influence disease outcomes without compromising crop performance. In commercial greenhouse production, nitrate is typically supplied at 19 mM NO_3_^−^. However, our findings indicate that restricting nitrate to a range of 3–5 mM NO_3_^−^ maintained flower biomass while enhancing metabolic defense capacity. These results suggest that optimizing nitrogen supply may serve as a complementary strategy to reduce reliance on chemical fungicides in ornamental production systems.

## Conclusions

This study shows that reduced nitrate availability enhances powdery mildew resistance in *Gerbera hybrida* and is associated with changes in primary and secondary metabolism. Reduced nitrate availability was accompanied by increased accumulation of polyketide- and phenylpropanoid-derived defense metabolites, including gerberin, gerberinside, parasorboside, and chlorogenic acid derivatives, while reducing nitrogen-containing primary metabolites. These findings suggest a shift in metabolic resources from nitrogen-rich primary metabolism toward carbon-based defense-related secondary metabolism. Moderate nitrate restriction enhanced resistance without compromising flower biomass, highlighting the potential of nitrogen management as a sustainable strategy for reducing reliance on chemical disease control in ornamental crop production. By linking nitrogen nutrition to defined metabolic pathways and disease outcomes, this study provides a physiological basis for integrating fertilization practices into powdery mildew management. Future studies integrating transcriptomics, enzyme activity analyses, and functional validation will be essential to determine whether nitrate-induced changes in polyketide metabolism directly contribute to powdery mildew resistance in *Gerbera hybrida*.

## Data Availability

The raw data supporting the conclusions of this article will be made available by the authors, without undue reservation.
