# Supplementary material for: Nitrate availability influences powdery mildew resistance and secondary metabolism in Gerbera hybrida
Source: Front Plant Sci. 2026 Aug 7;17:1879542. doi: 10.3389/fpls.2026.1879542 (PMC13493271; doi:10.3389/fpls.2026.1879542)
Supplement: Supplementary file 1 [file SupplementaryFile1.docx]

Secondary Metabolites at the Forefront: The Role of Nitrate in Shaping Powdery Mildew Resistance in *Gerbera hybrida*

Supplementary Material

**Supplementary Figure 1** Measured nitrate concentration in Trial 1. Average NO₃⁻ concentrations over the cultivation period were 1.7, 2.7, 6.0, 8.6, and 14.8 mM for 2 mM NO_3_^-^–19 mM NO_3_^-^, respectively.

**Supplementary Figure 2** Irrigation and drain in Trial 1. Electrical conductivity (EC) and pH remained similar across treatments throughout the cultivation period, with mean values of 2.3 dS m⁻¹ and 5.6, respectively. The average irrigation rate was 2.7 L m⁻² day⁻¹, with a mean drainage fraction of 65%.

**Supplementary Figure 3** Measured nitrate concentration in Trial 2. Average NO₃⁻ concentrations over the cultivation period were 2.8, 4.3, 6.4, and 8.9 mM for 3 mM NO_3_^-^–10 mM NO_3_^-^, respectively.

**Supplementary Figure 4** Irrigation and drain in Trial 2. Electrical conductivity (EC) and pH remained similar across treatments throughout the cultivation period, with mean values of 2.3 dS m⁻¹ and 5.5, respectively. The average irrigation rate was 2.4 L m⁻² day⁻¹, with a mean drainage fraction of 34%.

**Supplementary Figure 5** Heatmap of metabolites in gerbera leaves extracted in MeOD-D_2_O (1:1, v/v). Mean values are shown. (A) Trial 1, n = 18. (B) Trial 2, n = 15.

**Supplementary Figure 6** Bar plots showing integrated signals from non-identified metabolites. Different letters indicate a significant difference, p < 0.05, after applying the Benjamini–Hochberg correction. Mean values with standard error are shown. Trial 1, n = 18; Trial 2, n = 15. Description of the integrated unknown signals is provided in Supplementary Table 1.

**Supplementary Figure 7** PCA score plot of metabolites in gerbera leaves extracted in MeOD-D_2_O (1:1, v/v). n=15.

**TABLES**

**Supplementary Table 1** List of bins used in the study.

| **Metabolites** | **Beginning bin range (ppm)** | **End bin range (ppm)** |
| --- | --- | --- |
| TSP | -0.02 | 0.02 |
| Unknown1 | 0.85 | 0.9 |
| Unknown2 | 0.9327 | 0.942 |
| Unknown3 | 0.9776 | 0.9892 |
| Valine | 1.004 | 1.009 |
| Isoleucine | 1.009 | 1.014 |
| Unknown4 | 1.032 | 1.057 |
| 5-Hydroxyhexanoic acid 3-*O*-*β*-D-glucoside | 1.18 | 1.22 |
| Threonine | 1.33 | 1.346 |
| Parasorboside | 1.36 | 1.379 |
| Unknown5 | 1.392 | 1.4 |
| Unknown6 | 1.41 | 1.417 |
| Gerberine | 1.438 | 1.45 |
| Alanine | 1.467 | 1.476 |
| CGAderivative1 | 2.211 | 2.227 |
| Unknown7 | 2.885 | 2.9 |
| Asparagine | 2.913 | 2.945 |
| Ethanolamine | 3.1 | 3.134 |
| Unknown8 | 4.35 | 4.357 |
| Unknown9 | 4.357 | 4.364 |
| Glucose | 4.56 | 4.6 |
| Gerberinol | 5.889 | 5.915 |
| Unknown10 | 6.075 | 6.11 |
| Unknown11 | 6.206 | 6.243 |
| CGA derivative B | 6.25 | 6.27 |
| Chlorogenic acid | 6.338 | 6.353 |
| 5-*O*-Caffeoyl quinic acid | 6.385 | 6.4 |
| 5-*O*-Ferruloyl quinic aicd | 6.471 | 6.491 |
| Gerberinside | 7.25 | 7.296 |
